# Autophagy regulates the therapeutic potential of adipose-derived stem cells in LPS-induced pulmonary microvascular barrier damage

**DOI:** 10.1038/s41419-019-2037-8

**Published:** 2019-10-23

**Authors:** Chichi Li, Jingye Pan, Lechi Ye, Honglei Xu, Beibei Wang, Hanyan Xu, Lingna Xu, Tongtong Hou, Dan Zhang

**Affiliations:** 1 Plastic Surgery, The First Affiliated Hospital of Wenzhou Medical University, Nanbaixiang, Wenzhou City, Zhejiang Province 325000 PR China; 2 Department of Intensive Care Unit, The First Affiliated Hospital of Wenzhou Medical University, Nanbaixiang, Wenzhou City, Zhejiang Province 325000 PR China; 3 Department of Colorectal & Anal Surgery, The First Affiliated Hospital of Wenzhou Medical University, Nanbaixiang, Wenzhou City, Zhejiang Province 325000 PR China; 4 Emergency Department, The First Affiliated Hospital of Wenzhou Medical University, Nanbaixiang, Wenzhou City, Zhejiang Province 325000 PR China; 5 Department of Respiratory Medicine, The First Affiliated Hospital of Wenzhou Medical University, Nanbaixiang, Wenzhou City, Zhejiang Province 325000 PR China

**Keywords:** Cell biology, Stem cells

## Abstract

Adipose-derived stem cells (ADSCs) have been shown to be beneficial in some pulmonary diseases, and the paracrine effect is the major mechanism underlying ADSC-based therapy. Autophagy plays a crucial role in maintaining stem cell homeostasis and survival. However, the role of autophagy in mediating ADSC paracrine effects has not been thoroughly elucidated. We examined whether ADSCs participate in lipopolysaccharide (LPS)-induced pulmonary microvascular endothelial cell (PMVEC) barrier damage in a paracrine manner and illuminated the role of autophagy in regulating ADSC paracrine effects. PMVECs and ADSCs with or without autophagy inhibition were cocultured without intercellular contact, and the microvascular barrier function was assessed after LPS treatment. ADSC paracrine function was evaluated by detecting essential growth factors for endothelial cells. For in vivo experiments, ADSCs with or without autophagy inhibition were transplanted into LPS-induced lung-injury mice, and lung injury was assessed. ADSCs significantly alleviated LPS-induced microvascular barrier injury. In addition, ADSC paracrine levels of VEGF, FGF, and EGF were induced by LPS treatment, especially in the coculture condition. Inhibiting autophagy weakened the paracrine function and the protective effects of ADSCs on microvascular barrier injury. Moreover, ADSC transplantation alleviated LPS-induced lung injury, and inhibiting autophagy markedly weakened the therapeutic effect of ADSCs on lung injury. Together, these findings show that ADSC paracrine effects play a vital protective role in LPS-induced pulmonary microvascular barrier injury. Autophagy is a positive mediating factor in the paracrine process. These results are helpful for illuminating the role and mechanism of ADSC paracrine effects and developing effective therapies in acute lung injury.

## Introduction

Acute lung injury (ALI) remains a major contributor to high morbidity and mortality inside and outside the hospital. One of the major pathological features of this syndrome is that endothelial hyperpermeability leads to lung inflammation and injury^1^. Lipopolysaccharide (LPS, endotoxin) derived from gram-negative bacteria has been considered the principal eliciting factor in activating a number of inflammatory pathways to induce pulmonary endothelial permeability during the development of ALI^2^. In recent years, many studies have been established to explore and understand the pathogenesis of ALI; however, the therapeutic effect has not been notably enhanced, and the overall mortality remains high. Therefore, it is urgent to develop new therapeutics.

Stem cell therapy has become commonly accepted in recent years. Adipose-derived stem cells (ADSCs), a population of mesenchymal stem cells within adipose tissue, can be easily harvested and rapidly proliferated under minimal invasiveness conditions. Previous studies have demonstrated that ADSCs have superior anti-inflammatory and regeneration-enhancing properties because they can secrete more bioactive factors than bone marrow-derived stem cells (MSCs)^3^. Paracrine action but not transdifferentiation has been considered the predominant mechanism for the role of stem cells in protecting against tissue injury and promoting tissue restoration and repair. Increasing evidence suggests that ADSC transplantation is beneficial, especially in lung-related diseases^4,5^. However, the role and related mechanisms of ADSCs and their paracrine function in maintaining pulmonary microvascular barrier integrity under LPS-induced ALI have not been thoroughly elucidated to date.

Autophagy is a cellular self-degradative process by which damaged organelles and denatured proteins are sequestered in autophagosomes and delivered to lysosomes for eventual degradation. Basal autophagy serves crucial roles in maintaining cellular homeostasis and remodeling during normal development. Dysfunctional autophagy has been associated with a variety of pulmonary diseases, including ALI, pulmonary arterial hypertension, and chronic obstructive pulmonary disease^6–8^. Many recent studies have demonstrated the roles of some autophagy-related genes and related pathways in mediating the maintenance, proliferation, and differentiation of various stem cells^9,10^. However, whether autophagy mediates ADSCs to protect against pulmonary microvascular barrier injury under ALI remains elusive.

The goals of this study were to examine (1) the function of ADSCs cocultured with LPS-challenged PMVECs in maintaining pulmonary microvascular endothelial barrier integrity, (2) the therapeutic effect of transplanted ADSCs on LPS-induced ALI, and (3) the effects of autophagy on the function of ADSCs in pulmonary microvascular endothelial barrier damage and ALI under LPS treatment conditions.

## Materials and methods

### Chemicals and antibodies

LPS (from *Escherichia coli)* (L-2630) and FITC-dextran (53379) were purchased from Sigma-Aldrich. Endothelial cell growth medium (ECM, 1001) was purchased from ScienCell. Short hairpin RNA against autophagy-related gene 7 (sh*ATG7*) plasmids, ATG7 shRNA lentivirus particles and antibodies targeting ATG7 were purchased from Santa Cruz Biotechnology (sc-41448-SH, sc-41448-V, and sc-376212). The following antibodies, including anti-microtubule-associated protein 1-light chain 3 (LC3) B (3868), anti-sequestosome 1/p62 protein (p62) (5114), anti-GAPDH (2118) and anti-rabbit IgG (7074), were purchased from Cell Signaling Technology. mRFP-GFP-LC3 adenoviral vectors were obtained from Han Bio Technology Co. Ltd. (HanBio, Shanghai, China). Anti-zonula occludens-1 (ZO-1) and anti-claudin-5 were purchased from Abcam (ab216880 and ab131259). Rhodamine-conjugated phalloidin was purchased from Invitrogen (Molecular Probe, R415). The following enzyme-linked immunosorbent assay (ELISA) kits were purchased from R&D Systems: tumor necrosis factor (TNF)-α (MTA00B), interleukin (IL)-1β (MLB00C), IL-10 (M1000B), vascular endothelial growth factor (VEGF) (MMV00), fibroblast growth factor (FGF) (MFB00) and epidermal growth factor (EGF) (MEG00). Evans blue (E2129) was purchased from Sigma-Aldrich.

### Adipose-derived stem cell culture and autophagy inhibition

Mouse ADSCs (Cloud-Clone Corp., CSI031 Mu01) were cultured in ADSC complete growth medium. The primary ADSCs were harvested with trypsin when they had grown to approximately 80% confluence. Then, the cells were plated on the new culture dishes at ~6000 cells/cm^2^. In the present experiment, to determine whether autophagy could influence ADSC characteristics, we constructed ADSC lines with or without autophagy inhibition by applying shRNA targeting autophagy-related gene (ATG)7 and a lentiviral transfection assay. ADSCs^shRNA-ATG7^ and ADSCs^shRNA-Con^ represented ADSCs with and without autophagy inhibition, respectively (as detailed in the Supplementary materials and methods).

### Mouse pulmonary microvascular endothelial cell culture

For in vitro experiments, mouse pulmonary microvascular endothelial cells (PMVECs) (Cloud-Clone Corp., CSI027 Mu01) were cultured in endothelial cell medium. The cells were detached with trypsin/ethylene diamine tetra acetic acid when they grew to confluence (usually 3–5 d), and then they were transferred to new dishes at a split ratio of 1:2 for further propagation. In this study, PMVECs of passages 3–5 were selected for analysis.

### In vitro cell grouping and LPS challenge

In this study, PMVECs were divided into four groups as follows: PMVECs, LPS-challenged PMVECs, LPS-challenged PMVECs cocultured with ADSCs^shRNA-Con^ and LPS-challenged PMVECs cocultured with ADSCs^shRNA-ATG7^. In the coculture system, PMVEC suspensions were added to the top chambers of the cell-culture inserts (0.4-μm pore size polyester membrane from Corning, Inc.) at a density of 50,000 cells per insert well, and ADSCs were cultured on the bottom at a density of 70,000 cells/well, resulting in coculturing without direct contact. To mimic LPS-induced lung injury, PMVECs were incubated in Dulbecco’s modified Eagle’s medium/F12 supplemented with 10% fetal bovine serum containing 100 ng/ml LPS and cultured for 6 h. In addition, culture media from LPS-treated PMVECs and normal cultured PMVECs were collected as LPS-treated PMVECs-culture media (CM) and PMVECs- CM, then these media were added into ADSCs culture media, so that to verify the effect of PMVECs on ADSCs autophagy in cocultured system.

### Collection of culture media from ADSCs

When ADSCs had grown to 70–80% confluence, they were washed by PBS and then incubated in serum-free Dulbecco’s modified Eagle’s medium. These cells were separated into two groups, one group of cells was treated with 100 ng/ml LPS and another group of cells was treated with equal volume of PBS for 6 h. After that, the media were collected as LPS-treated ADSCs-CM and ADSCs-CM. These media were added into PMVECs cultured media, so that to make clear the paracrine function of ADSCs on LPS-induced pulmonary microvascular injury.

### Detecting ADSC autophagy through the mRFP-GFP adenoviral vector

Adenoviral transfection was carried out when ADSCs reached ~50–70% confluence. The cells were incubated in growth medium with the adenoviruses at an MOI of 100 for 2 h at 37 °C. The cells were grouped as follows: ADSCs, ADSCs cocultured with PMVECs, ADSCs with direct LPS treatment, and ADSCs cocultured with LPS-challenged PMVECs. Using the mRFP-GFP-LC3 adenoviral vectors, LC3B-positive, neutral pH autophagosomes, and acidic pH autolysosomes could be detected as green fluorescence (GFP) and red fluorescence (RFP), respectively. Autophagic structures were detected under a Nikon A1 R laser confocal microscope. Autophagic flux was assessed by evaluating the ratio of yellow puncta to the total number of GFP and mRFP puncta.

### Protein preparation and immunoblotting

ADSCs or PMVECs were homogenized in RIPA lysis buffer, and then the homogenate was incubated on ice for 45 min and centrifuged at 4 °C (12,000 rpm for 5 min). After determining the protein concentration using the BCA protein assay kit according to the manufacturer’s instructions, the supernatant was collected and the proteins were separated in sodium dodecyl sulfate-polyacrylamide gels (SDS-PAGE) at 120 V for 2 h. The proteins in the gels were transferred onto a polyvinylidene difluoride membrane, which was then incubated with specific primary antibodies, followed by incubation with horseradish peroxidase-conjugated secondary antibodies for 1 h. Finally, protein visualization was performed using Pierce ECL western blotting substrate and autoradiography. The following primary antibodies were used: anti-LC3B, anti-p62, anti-ATG7, anti-ZO-1, anti-claudin-5, and anti-GAPDH. Quantity One 4.6 software was applied to analyze the blots, and the data were normalized to GAPDH and expressed as the optical density (OD) integration.

### F-actin labeling

We determined stress fiber formation by detecting F-actin using a rhodamine-conjugated phalloidin molecular probe according to the manufacturer’s instructions. Cells that were treated with 100 ng/ml LPS were fixed with 3.7% paraformaldehyde for 10 min, permeabilized with 0.5% Triton X-100, and finally stained with rhodamine-conjugated phalloidin. The nuclei were labeled with 4′,6-diamidino-2-phenylindole (DAPI). The labeled cells were detected under a Nikon A1 R laser confocal microscope. We quantified F-actin conformation by analyzing the percentage of cells containing stress fiber in each experimental group.

### Transendothelial permeability assay

In the transwell system, PMVECs and ADSCs were cultured in the upper and lower wells, respectively. FITC-dextran (1 mg/ml, MW 40,000) was added on top of the wells, allowing it to permeate through the PMVEC monolayers. After LPS treatment, the media was collected at 0 and 6 h from the lower compartments of the transwell chambers, and then an equal volume of cell basal medium was refilled. The fluorescence values of FITC-dextran in the upper and lower wells were determined with a fluorescence microplate reader (BioTek Instruments, Inc., FLX800TBID) at an excitation wavelength of 492 nm and a detecting emission of 520 nm. The final values were calculated by subtracting the fluorescence values at 0 h. The results are presented as the percentage of the fluorescence values in the control group.

### Terminal deoxynucleotidyl transferase-mediated dUTP nick end labeling (TUNEL)

The PMVECs were fixed with 4% paraformaldehyde at room temperature for 30 min. This was followed by several rinses in PBS and permeabilization in 0.1% Triton X-100 in 0.1% sodium citrate on ice for 2 min. Then, 50 μl of TUNEL staining solution was added to the wells, and the cells were incubated at 37 °C in a dark humidified chamber for 1 h. Finally, the cells were incubated with DAPI for 20 min at room temperature. After that, apoptotic cells were detected by fluorescence microscopy (×200 , excitation wavelength: 488 nm, emission wavelength: 530 nm), and the apoptotic rate was analyzed. The cells containing green fluorescence dots were defined as apoptotic cells.

### Detection of apoptosis by flow cytometry

Apoptosis was further detected using the Annexin V-FITC apoptosis detection kit according to the manufacturer’s instructions, and cells were analyzed using a flow cytometer. Briefly, PMVECs were digested with 0.25% trypsin and then rinsed twice with PBS. Then, the cells were resuspended in 1× binding buffer at a concentration of 1 × 10^6^ cells/ml, and 100 µl of the solution was transferred to 5-ml culture tubes followed by the addition of 5 µl Annexin V-FITC and 5 µl propidine iodide. The resulting solution was incubated at room temperature in the dark for 15 min followed by the addition of 400 µl 1 × binding buffer. Apoptotic rates were analyzed immediately by flow cytometry (FCM) (BD Biosciences, FACS Aria III).

### ELISA

The medium from the coculturing system was collected and centrifuged at 4 °C (1000 rpm for 15 min). The supernatant was collected, and concentrations of VEGF, FGF, and EGF in the medium were determined using mouse VEGF, FGF and EGF ELISA kits according to the manufacturer’s instructions.

### Quantification of mRNA using real-time reverse-transcription polymerase chain reaction (RT-PCR)

Real-time RT-PCR was performed to determine relative mRNA levels of VEGF, FGF, and EGF in ADSCs and cocultured PMVECs. Total RNA was isolated from ADSCs and cocultured PMVECs by using a GenElute Mammalian Total RNA Kit (Sigma, St. Louis, MO) according to the manual. The purified RNA was used for reverse transcription using One Step RT-PCR (TaKaRa). Primers used in the PCR reaction were as follows: VEGF, forward: 5′-AACGATGAAGCCCTGGAGTG-3′ and reverse: 5′-TGAGAGGTCTGGTTCCCGA-3′; FGF, forward: 5′-CCTCTCAGAGACCTACGTTCAA-3′ and reverse: 5′-GGAGGTCAAGGCCACAAT-3′; EGF, forward: 5′-GCTGTGACGGTCCTTACAATG-3′; reverse: 5′-CAGTTCCCACCACTTCAGGTC-3′ or GAPDH: forward: 5′-CGACTTCAACAGCAACTCCCACTCTTCC-3′ and reverse: 5′-TGGGTGGTCCAGGGTTTCTTACTCCTT-3′. The cycle conditions were: 48 °C for 30 min and 94 °C for 1 min, followed by 40 cycles at 94 °C for 15 s, 65 °C for 30 s and at 68 °C for 1 min. Polymerase chain reaction products were detected with 2% agarose gel electrophoresis and analyzed with a gel imaging analyzer. The ratio of the target gene (VEGF, EGF and EGF) OD to the reference gene (GAPDH) OD in the same sample was calculated and considered as the target gene mRNA relative content. All the assays were performed in duplicate.

### In vivo model of ALI and study grouping

In the in vivo experiments, 8–10-week-old wild-type BALB/c mice (Animal Center, Fudan University, Shanghai, China) were used. The animals were starved of solid food but had free access to water 12 h before the experiments. All experimental protocols were approved by the Committee of Animal Care Fudan University. All animals were handled in compliance with the Guideline for the Care and Use of Laboratory Animals^11^. The mice were anesthetized and then orally intubated with a sterile plastic catheter and challenged by intratracheal instillation of 2 mg of LPS kg^−1^ b.w. ADSCs^shRNA-ATG7^ or ADSCs^shRNA-Con^ (2.5 × 10^5^ cells, 100 μl total volume) were slowly infused via a caudal venous canula 30 min following LPS challenge. The mice were sacrificed 48 h after LPS instillation.

### Assessment of histologic lung damage

The mice were humanely killed after LPS challenge, and fresh left lung tissues were harvested and immediately fixed in 10% formalin, embedded in paraffin, and cut into 4-μm thick paraffin sections. The sections were stained with hematoxylin and eosin (H&E). Lung injury was assessed according to four categories: interstitial inflammation, neutrophil infiltration, congestion, and edema. The assessment results are shown as scores on a 0 to 4 point scale: no injury = score of 0; injury in 25% of the field = score of 1; injury in 50% of the field = score of 2; injury in 75% of the field = score of 3; and injury throughout the field = score of 4^12^. Ten microscopic fields from each slide were analyzed. The sums of the tissue slides were averaged to evaluate the severity of lung injury. All microscopic sections were scored by a pathologist who was blinded to the experimental groups and protocol.

### Pulmonary edema detection

The severity of lung edema was assessed by determining the ratio of the wet/dry lung weight. Briefly, after measuring the lung wet weight, the same lung was dried at 56 °C for 72 h, and the ratio of the wet/dry lung weight was then calculated.

### Measurement of lung vascular permeability by intravenous injection of Evans blue dye

Lung microvascular barrier permeability was assessed using Evans blue dye. The mice were intravenously injected with 20 mg kg^−1^ dye 30 min before the termination of LPS treatment. All mice were euthanized after LPS challenge, and thoracotomies were performed. The lungs were perfused free of blood with physiological saline containing 500 units of heparin at 5 cm H_2_O. The dried lung tissues were immersed in 0.5 ml formamide and homogenized. Then, the homogenate was incubated at 37 °C for 24 h and centrifuged at 5,000 g for 30 min. The optical density of the supernatant was spectrophotometrically measured at 620 nm. The total amount of Evans blue was determined by comparison to standard absorbance curves.

### Collection of bronchoalveolar lavage fluid (BALF) and detection of cytokines by ELISA

For the retrieval of BALF, the left general bronchus of each mouse was ligated, and then the right lung was irrigated with 0.5 ml PBS after LPS challenge. The fluid from three times of lavage was pooled. The total BALF from each mouse was centrifuged at 4 °C (1000 rpm for 15 min). The concentrations of TNF-α, IL-1β, and IL-10 in BALF supernatant were determined using mouse TNF-α, IL-1β, and IL-10 ELISA kits according to the manufacturer’s instructions.

### Statistical analyses

Data were obtained from at least three separate experiments performed in triplicate. SPSS 13.0 software was used for data processing. The results are shown as the mean and standard deviation (mean ± SD). Differences between groups were analyzed by one-way analysis of variance and post hoc Bonferroni correction for multiple comparisons. The histologic semiquantitative analysis was compared by the nonparametric Mann–Whitney test. A *P-*value < 0.05 was considered to be statistically significant.

## Results

### PMVEC coculturing affected ADSC autophagy

**Fig. 1:**
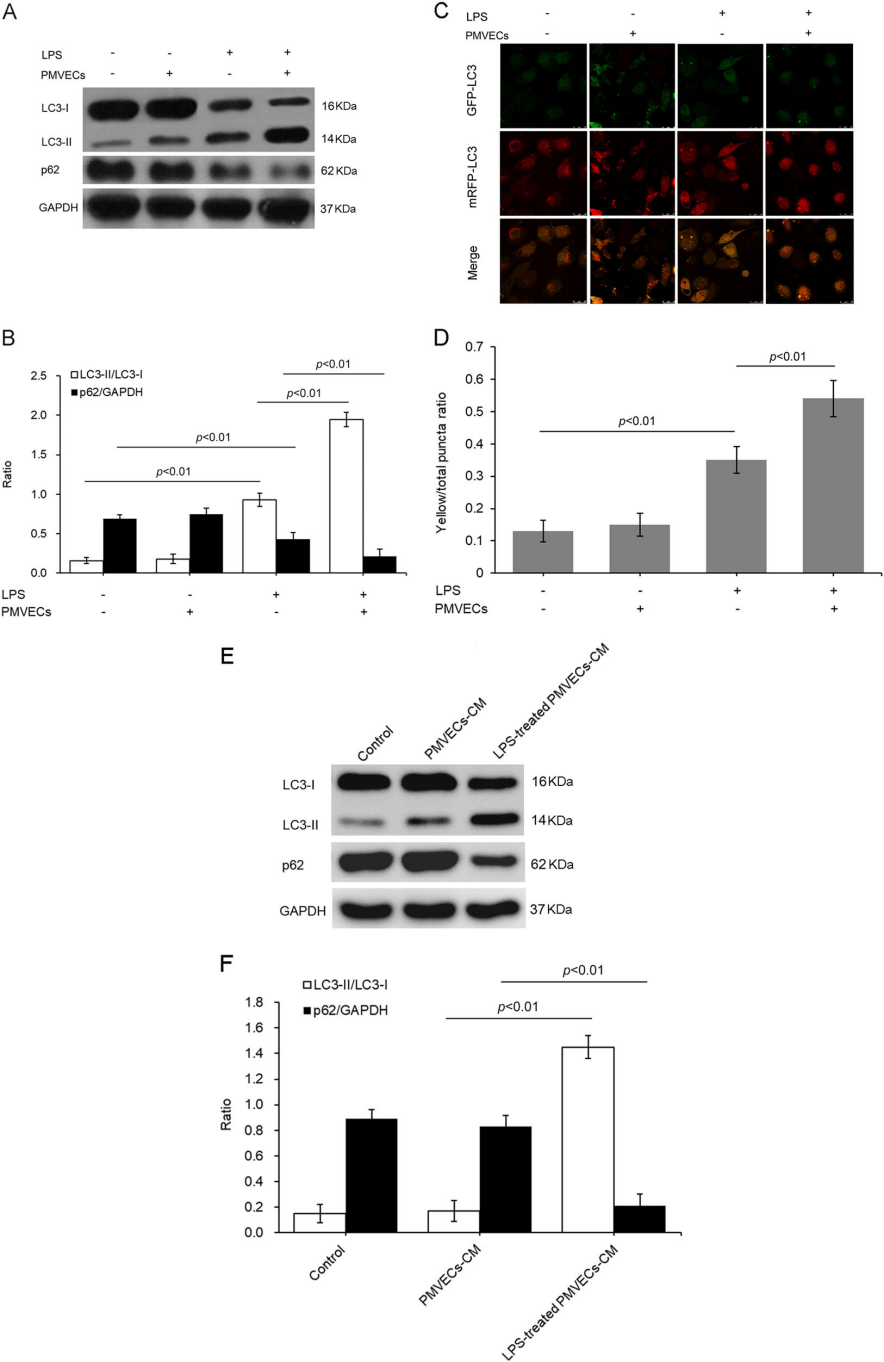
Cocultured PMVECs increased the autophagy level of ADSCs. **a** Representative western blot image of LC3 and p62 in ADSCs. **b** Statistical analysis of the expressions of LC3 and p62. **c** Representative images of ADSCs transiently overexpressing mRFP-GFP-LC3. The neutral pH LC3B-positive autophagosomes (green fluorescence) and acidic pH LC3B-positive autolysosomes (red fluorescence) were detected using a confocal microscope. **d** Quantification of mRFP^+^-GFP^+^ (yellow) puncta in at least 10 cells per group. **e** Representative western blot image for LC3 and p62 in ADSCs treated by PMVECs-culture media (CM) or LPS-treated PMVECs-CM. **f** Statistical analysis of the expressions of LC3 and p62. The results are presented as the mean ± SD (*n* = 3)

To determine whether ADSCs underwent autophagy during LPS-induced pulmonary microvascular barrier damage, we detected the autophagy level of ADSCs in different experimental groups. As shown, we detected an increase in the expression of LC3-II but a decrease in that of p62 in the LPS treatment group. These effects were further enhanced when the cells were cocultured with LPS-challenged PMVECs (Fig. 1a, b).

Increased LC3-II levels can be related to augmented autolysosome formation or impaired clearance of autophagosomes. To differentiate between the abovementioned possibilities, we transfected Ad-mRFP-GFP-LC3, a specific marker for autophagosomes and autolysosomes, into ADSCs. We detected that the cells treated with LPS or cocultured with LPS-challenged PMVECs showed a typical accumulation of GFP-LC3 and mRFP-LC3 puncta in both the cytoplasm and perinuclear region (Fig. 1c). The number of mRFP-GFP-LC3 puncta in LPS-treated cells was much greater than that in the control cells, and the number of puncta was largest in the ADSCs cocultured with LPS-triggered PMVECs (Fig. 1d).

To further clarify the effect of cocultured PMVECs on ADSC autophagy, we detected the expression of LC3 and p62 in ADSCs treated with cultured media from PMVECs. The autophagy level was highest in the cells treated with the media from LPS-triggered PMVECs. The media from normal cultured PMVECs did not significantly affect the autophagy level of ADSCs (Fig. 1e, f).

### Modulation of ADSC autophagy affected the permeability and the expression of tight junction-associated proteins of LPS-treated PMVECs

We successfully constructed ADSC lines with or without autophagy inhibition using shRNAs targeting ATG7 or control shRNAs that were referred to as ADSCs^shRNA-ATG7^ and ADSCs^shRNA-Con^, respectively (Fig. S1a–c). Flow cytometry analysis demonstrated that ADSCs with or without autophagy inhibition had similar surface markers: CD90 (+), CD105 (+), CD44 (+) and CD45 (−) (Fig. S2).

**Fig. 2:**
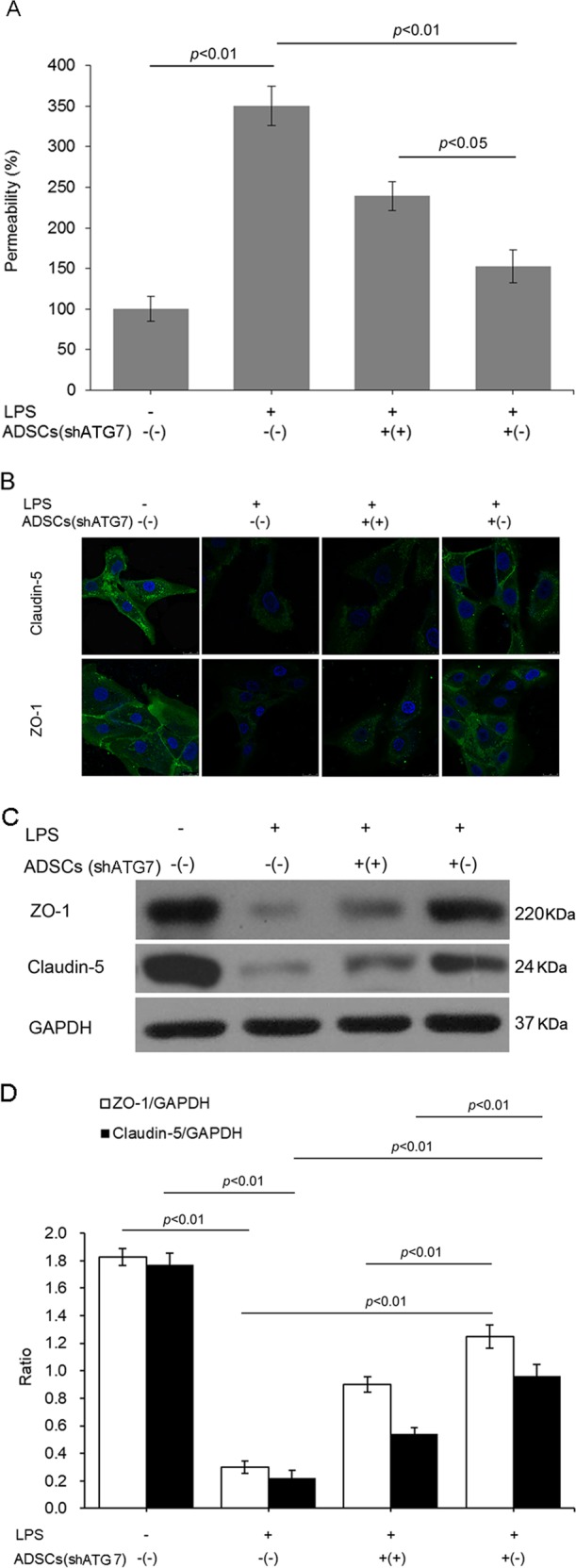
Modulating autophagy affected the permeability and tight junction-associated protein expression of LPS-treated PMVECs. **a** Permeability of PMVECs was detected through transwell assay. **b** Fluorescent images of ZO-1 and claudin-5 in PMVECs cocultured with ADSCs^shRNA-ATG7^ or ADSCs^shRNA-Con^. **c** Representative western blot image of ZO-1 and claudin-5 in PMVECs. **d** Statistical analysis of the expression of ZO-1 and claudin-5 in PMVECs. The results are presented as the mean ± SD (*n* = 3)

To determine whether modulating autophagy affected the regulatory function of ADSCs on PMVEC barrier integrity, we detected endothelial permeability in different culture conditions. Cocultured ADSCs with or without autophagy inhibition had no significant effect on the permeability of normally cultured PMVECs (Fig. S3). LPS treatment significantly increased PMVEC permeability, which was effectively lowered by coculturing with ADSCs^shRNA-Con^. The permeability in the group of PMVECs cocultured with ADSCs^shRNA-ATG7^ was much higher than that in the PMVECs cocultured with ADSCs^shRNA-Con^ (Fig. 2a).

To evaluate the effect of ADSC on LPS-induced tight junction injury, we further evaluated the expressions of ZO-1 and claudin-5, two vital constituent proteins in PMVEC tight junctions. As shown, LPS treatment destroyed the tight junction integrity and inhibited the expressions of both ZO-1 and claudin-5 in PMVECs. The injury to the tight junction integrity was improved, and the decrease in ZO-1 and claudin-5 was attenuated by ADSCs^shRNA-Con^ coculture. However, autophagy inhibition weakened the protective effect of ADSCs on tight junctions (Fig. 2b). Western blot analysis was applied to quantify the expression of ZO-1 and claudin-5. Consistent with the aforementioned findings, LPS challenge significantly inhibited the expression of ZO-1 and claudin-5 in PMVECs. However, the reduced expression of both proteins was effectively attenuated by coculturing with ADSCs^shRNA-Con^. Autophagy inhibition limited the protective effect of ADSCs on ZO-1 and claudin-5 expression (Fig. 2c, d).

### Modulation of ADSC autophagy affected stress fiber formation in PMVECs under LPS treatment

**Fig. 3:**
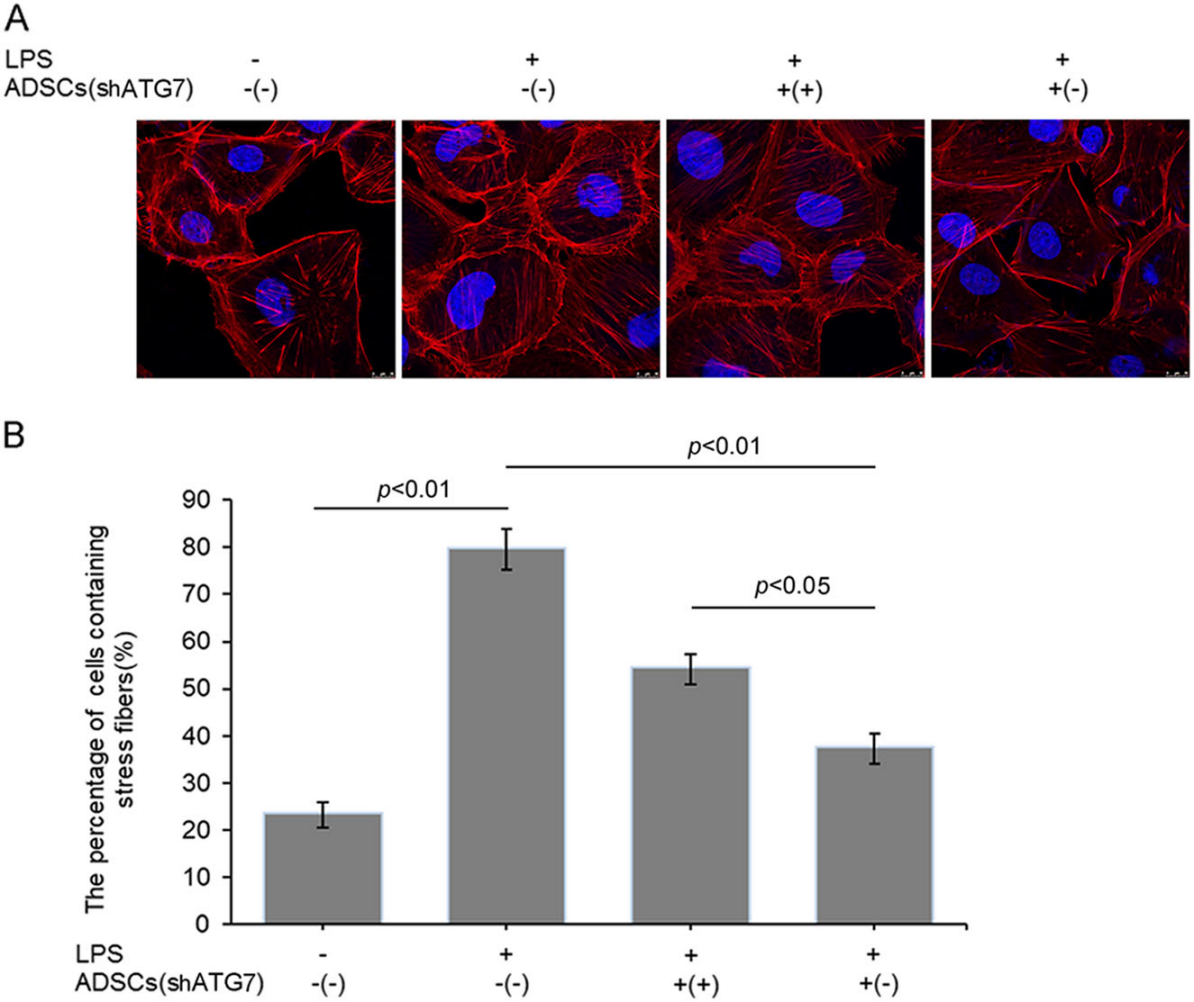
Modulating autophagy affected the role of ADSCs in PMVEC stress fiber formation under LPS-treated condition. **a** Stress fiber formation was detected with F-actin staining, nuclei were stained with DAPI. **b** Statistical analysis of the proportion of cells containing stress fiber (*n* = 3)

Cell–cell junctions are regulated by cortical F-actin stress fibers, which can be induced to polymerize to form transcytoplasmic cables. The cables generate tension between cell–cell junctions and focal adhesions in the extracellular matrix to disrupt cell–cell junctions and lead to increased paracellular permeability^13^. In the present study, actin stress fiber formation in PMVECs was significantly increased by LPS, but it was significantly attenuated when the cells were cocultured with ADSCs^shRNA-Con^. However, prohibiting autophagy by shRNA-ATG7 weakened the inhibitory effect of ADSCs on LPS-induced stress fiber formation in PMVECs (Fig. 3a). We further quantified the cells with stress fibers in different groups. LPS treatment remarkably increased the proportion of cells containing stress fibers. Cocultured ADSCs with or without autophagy inhibition effectively decreased the percentage of cells containing stress fibers. However, autophagic inhibition, in part, reduced the effect of ADSCs on the formation of stress fibers in PMVECs (Fig. 3b).

### Inhibition of autophagy weakened the inhibitory action of ADSCs on LPS-triggered PMVEC apoptosis

**Fig. 4:**
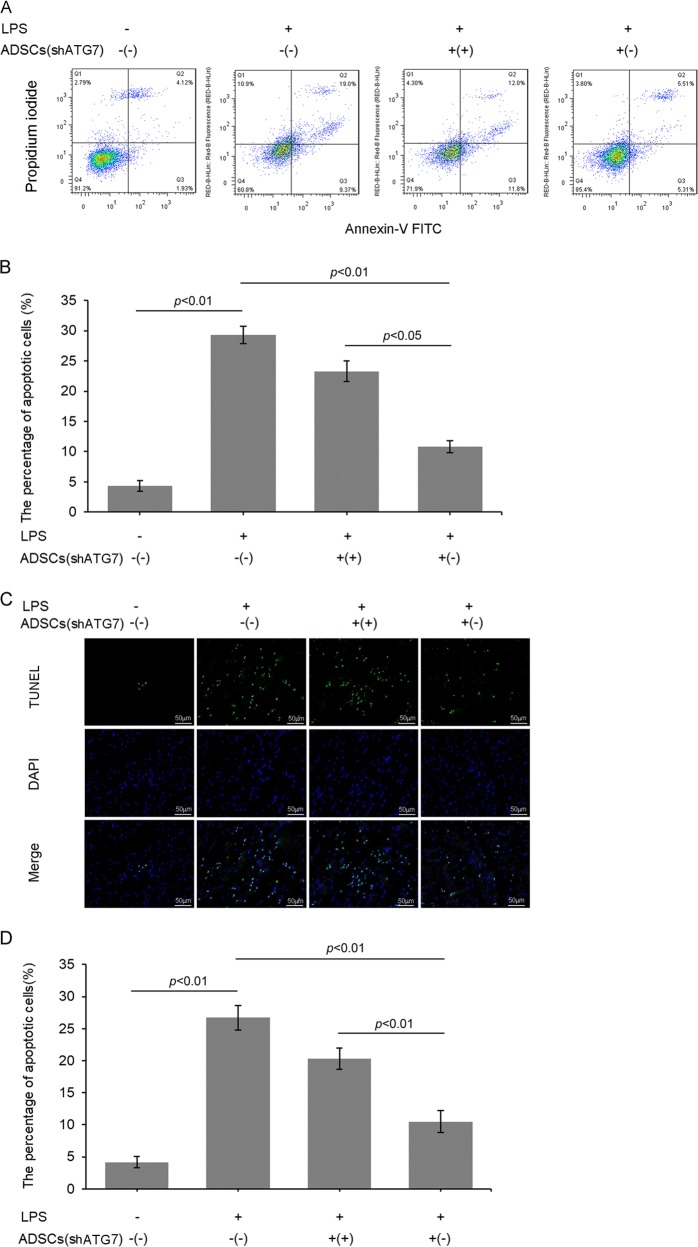
Inhibition of autophagy weakened the inhibitory action of ADSCs on LPS-triggered PMVEC apoptosis. **a**, **b** Typical flow cytometry quadrant diagrams and corresponding statistical data for apoptotic PMVECs. The top left, top right, and bottom right plots represent the necrotic cells and the late and early apoptotic cells, respectively. **c**, **d** Representative fluorescence images and corresponding statistical analysis of apoptosis by TUNEL staining. The results are presented as the mean ± SD (*n* = 3)

Apoptosis is one of the major causes of LPS-induced endothelial injury. In this study, we examined the effects of LPS on PMVEC apoptosis and whether it might be affected by ADSCs. In one experiment, we examined changes in the apoptosis rate by FCM. LPS treatment increased the PMVEC apoptosis rate from 4.35 ± 0.87% to 29.32 ± 1.45%. ADSCs^shRNA-Con^ coculture decreased the LPS-induced PMVEC apoptosis rate to 10.81 ± 1.01%, whereas ADSCs^shRNA-ATG7^ only lowered the apoptotic index to 23.34 ± 1.66% (Fig. 4a, b).

In another experiment, the TUNEL assay was applied to measure apoptosis levels. The apoptosis rate of LPS-treated cells was significantly increased (26.72 ± 1.88%) and was decreased in PMVECs cocultured with ADSCs^shRNA-Con^ (10.52 ± 1.69%) and in PMVECs cocultured with ADSCs ^shRNA-ATG7^ (20.34 ± 1.67%) compared with that of blank control cells (4.17 ± 0.89%) (Fig. 4c, d).

### Regulation of autophagy affected the treatment efficiency of ADSCs in LPS-induced mouse lung injury

**Fig. 5:**
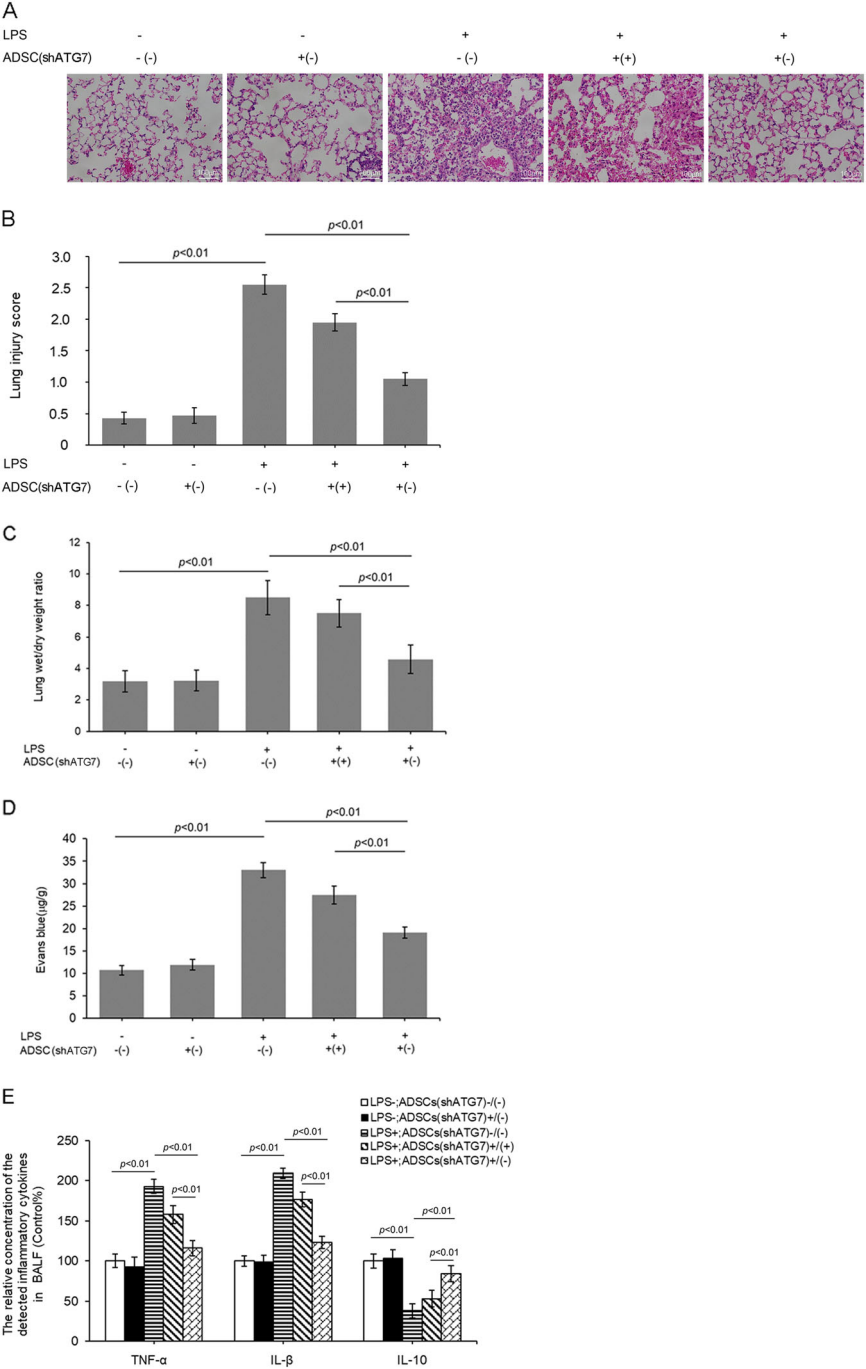
Autophagy affected the treatment efficacy of ADSCs in LPS-induced mouse lung injury. **a** Representative lung histopathological slices stained with hematoxylin and eosin (H&E). **b** Microscopic injury of the lungs was statistically scored. **c** Lung water content was determined by calculating the lung wet/dry weight ratio. **d** Assessment of lung microvascular permeability by detecting the content of Evans blue per gram of lung tissue. **e** Concentrations of tumor necrosis factor (TNF)-α, interleukin (IL)-1β and IL-10 in bronchoalveolar lavage fluid (BALF) from LPS-treated mouse lungs. The results are presented as the mean ± SD (*n* = 24/group, 6 for H&E staining and pathological scores; 6 for wet/dry weight ratio; 6 for the detection of Evans blue content; 6 for BALF collection and enzyme-linked immunosorbent assay)

The histological assessment of in vivo analyses revealed that LPS stimulated a striking influx of polymorphonuclear leukocytes into the alveolar space, as well as marked congestion and edema in the lung tissue. Transplanted ADSCs^shRNA-Con^ effectively attenuated LPS-triggered lung injury. However, the protective effect of transplantation of ADSCs^shRNA-ATG7^ was much worse than that of ADSCs^shRNA-Con^ (Fig. 5a). Through semiquantitative analysis of lung sections using lung injury scores, we found that LPS-induced lung injury was attenuated by transplantation of ADSCs^shRNA-Con^, and inhibition of ATG7 expression markedly weakened the therapeutic efficacy of ADSCs in LPS-induced lung injury (Fig. 5b).

In another experiment, we evaluated cell apoptosis in pulmonary tissues. LPS induced significant cell apoptosis, which was effectively attenuated by transplantation of ADSCs^shRNA-Con^. Inhibition of ATG7 expression, however, markedly weakened the efficacy of ADSCs in LPS-induced cell apoptosis (Fig. S4).

In addition, lung edema was assessed by detecting the lung wet/dry weight ratio. LPS challenge induced a significant increase in the lung wet/dry weight ratio. ADSC transplantation attenuated pulmonary vascular leakage and lung edema in LPS-challenged mice. However, the inhibitory action of ADSCs on LPS-induced lung edema was markedly weakened by ATG7 inhibition (Fig. 5c).

Furthermore, a leak index was determined using Evans blue dye to further elucidate the severity of lung vascular leakage. Evans blue leakage was significantly increased in the LPS group, and it was significantly attenuated by ADSC transplantation. However, shRNA-ATG7 intervention significantly reduced the protective effect of ADSCs against LPS-induced lung edema (Fig. 5d).

### Regulation of autophagy modulated the effects of ADSCs on pulmonary inflammation in mice subjected to LPS

An excessive inflammatory reaction is one of the causes of LPS-associated ALI. To evaluate the inflammatory reaction, we measured the levels of some cytokines that mediate inflammation in BALF. LPS significantly increased the production of the proinflammatory cytokines TNF-α and IL-1β but decreased that of the anti-inflammatory cytokine IL-10. Transplantation of ADSCs^shRNA-Con^ attenuated the production of TNF-α and IL-1β but effectively promoted the production of IL-10. Prior inhibition of autophagy weakened the ability of ADSCs to regulate the production of these inflammatory cytokines (Fig. 5e).

### ADSCs paracrine protected against LPS-induced PMVECs injury

**Fig. 6:**
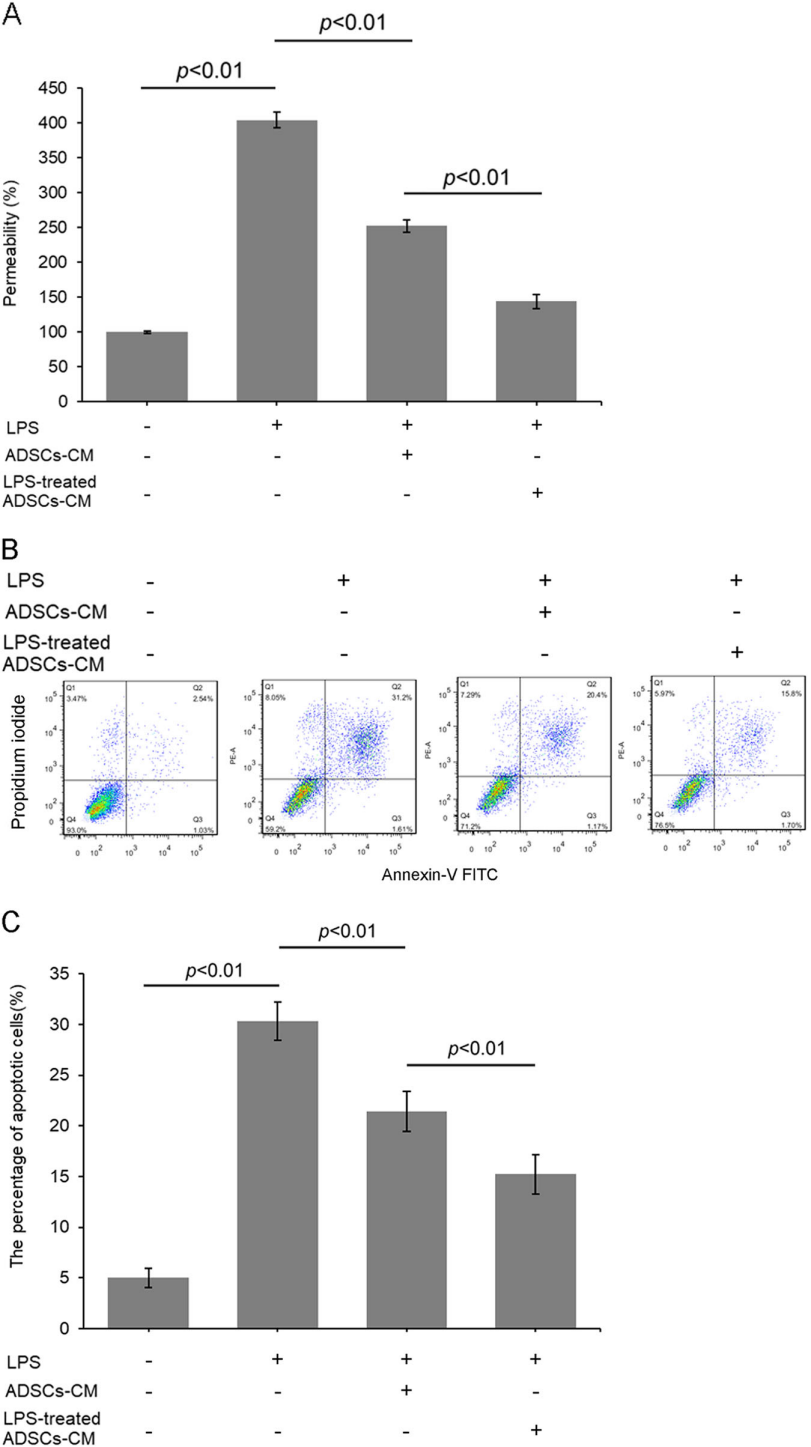
The effects of ADSCs paracrine on LPS-induced pulmonary microvascular injury. **a** Permeability of HPMVECs was detected through transwell assay. **b**, **c** Typical flow cytometry quadrantal diagrams and corresponding statistical data for apoptotic PMVECs

To clarify whether ADSC paracrine effects affected the LPS-induced PMVEC permeability, we applied cultured media from ADSCs to PMVECs. Media from individually cultured ADSCs effectively alleviated PMVEC permeability. In comparison, media from LPS-treated ADSCs showed more remarkable efficacy in decreasing LPS-induced PMVEC permeability (Fig. 6a).

The paracrine effects of ADSCs on PMVEC apoptosis was demonstrated by treating PMVECs with cultured media from ADSCs. Cultured media from ADSCs effectively alleviated PMVEC apoptosis. However, compared with media from control ADSCs, media from LPS-preconditioned ADSCs showed a more significant effect on inhibiting LPS-induced PMVEC apoptosis (Fig. 6b, c).

### Paracrine effects of ADSCs were mediated by autophagy

**Fig. 7:**
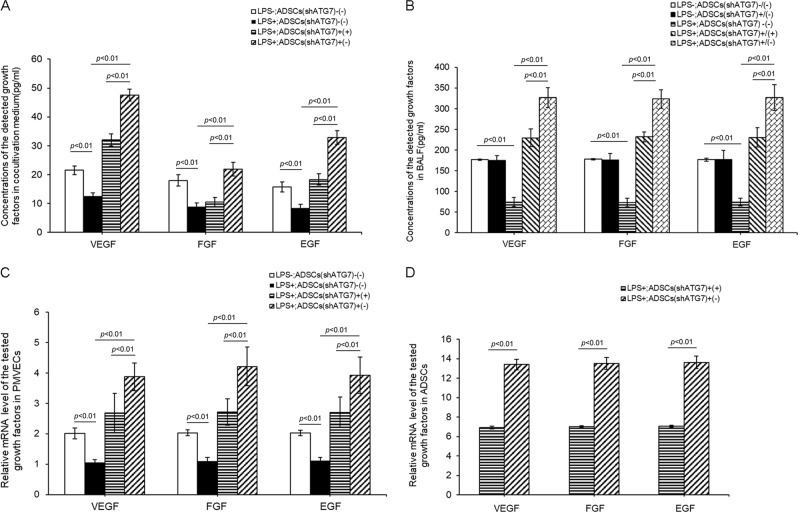
Autophagy mediates ADSCs paracrine. **a** Concentrations of VEGF, FGF and EGF in the coculturing medium of ADSCs and PMVECs under LPS treatment were detected. **b** Concentrations of VEGF, FGF and EGF in BALF from experimental mice were detected. **c**, **d** mRNA expressions of VEGF, FGF, and EGF in ADSCs and PMVECs under coculturing condition. Results are mean ± SD (*n* = 3)

Some growth factors, such VEGF, FGF, and EGF, are essential for sustaining endothelial cell survival and growth. Previous studies have shown that ADSCs can produce and secrete VEGF, FGF and EGF, and these factors then exert paracrine effects^14,15^. To determine whether autophagy could influence the paracrine action of ADSCs, we detected the concentration of the three growth factors in the culture media of the different groups. Under normal culture conditions, low concentrations of VEGF, FGF, and EGF were detected in the medium containing only PMVECs. Under normal culture conditions, cocultured ADSCs ^shRNA-Con^ or ADSCs ^shRNA-ATG7^ had no significant effect on the levels of VEGF, FGF and EGF in the coculture medium (Fig. S5). LPS treatment reduced the concentration of the aforementioned growth factors. ADSCs cocultured with LPS-treated PMVECs remarkedly increased the levels of VEGF, FGF and EGF in the coculture medium. However, inhibition of autophagy affected the release of growth factors in cocultured ADSCs (Fig. 7a). In an in vivo experiment, we assessed the concentrations of VEGF, FGF, and EGF in BALF from different mouse groups. The concentrations of the aforementioned growth factors from LPS-treated lung tissue BALF were the lowest, and they were effectively improved by transplanted ADSCs. However, inhibition of autophagy weakened the promoting effect of ADSCs on growth factor levels in LPS-challenged lung BALF (Fig. 7b).

To clarify the origin of VEGF, FGF, and EGF in the coculture system, we quantified the mRNA expression in ADSCs and cocultured PMVECs. LPS treatment lowered the mRNA expression of the aforementioned growth factors in PMVECs, and ADSC coculture promoted mRNA expression in PMVECs. However, this effect of ADSCs was significantly inhibited by autophagy inhibition. Similarly, in the coculturing system, the mRNA expression of the three growth factors in wildtype ADSCs was significantly higher than that in autophagy-inhibited cells. In comparison to those in PMVECs, mRNA expression levels of the aforementioned growth factors were much higher in cocultured ADSCs. Therefore, ADSC paracrine effects play a more primary role than PMVEC autocrine effects, although PMVEC autocrine effects and ADSC paracrine effects co-occur in LPS-treated coculturing conditions (Fig. 7c, d).

## Discussion

Although ADSCs have been shown to have a protective role in ALI, whether autophagy participates in mediating the effects of ADSCs on LPS-induced lung microvascular barrier damage remains unknown. Our in vitro results showed that LPS-induced PMVEC microvascular barrier damage was alleviated by ADSC coculture. Our data demonstrated that ADSCs themselves had undergone improved autophagy in response to LPS treatment and that inhibiting autophagy weakened the effects of ADSCs on PMVEC microvascular barrier integrity. In vivo data showed that ADSC transplantation effectively lowered LPS-induced lung injury, as reflected by the alleviated microvascular leakage and inflammatory response. However, autophagy inhibition limited the protective action of ADSCs on ALI. Based on the vital role of ADSC paracrine effects in resisting tissue damage, we detected the effect of autophagy on the paracrine effect of ADSCs cocultured with LPS-treated PMVECs. We found that inhibiting autophagy reduced ADSC secretion of VEGF, FGF, and EGF. Our findings suggest that autophagy contributes to the inhibitory effect of ADSCs on LPS-induced damage in pulmonary microvascular endothelial cells, at least in part by positively regulating growth factor secretion, to maintain the integrity of the lung microvascular barrier.

Under LPS treatment conditions, one of the main causes of high pulmonary microvascular permeability is injury to tight junctions^16,17^. In the present study, LPS treatment inhibited the expression of both ZO-1 and claudin-5, two essential participants in the PMVEC tight junction complex. ADSC coculture partly inhibited the decreased expression of ZO-1 and claudin-5, which may be attributed to the paracrine function of ADSCs. Paracrine factors of ADSCs, especially some growth factors, have been reported to regulate the integrity and function of cell junctions. FGF-associated signaling plays a vital role in maintaining the integrity of endothelial adherens and tight junctions. Blocking FGF signaling dissociates the VE-cadherin/p120-catenin complex and then disassembles adherens and tight junctions, thus inducing impairment of endothelial barrier function^18^. Another study reported that treatment with IGF-I significantly decreased the permeability of brain microvascular endothelial cells exposed to OGD reoxygenation^19^. Although the role of VEGF and EGF in increasing endothelial permeability has been validated, the protective role of FGF in maintaining tight junctions may partly counteract this phenomenon. In this study, we found increased concentrations of FGF in the coculture media of ADSCs and PMVECs. In addition, ATP is vital in maintaining the integrity of endothelial cell junctions^20^, and some researchers have reported critical roles of some growth factors in mediating tricarboxylic acid cycle activity^21^, which is essential for ATP production. These findings support the importance of ADSC paracrine growth factors in maintaining the integrity of endothelial cells.

Endothelial cell apoptosis is another important cause of LPS-induced endothelial cell barrier damage. Consistent with a previous study, our findings revealed that ADSC coculture attenuated the apoptosis ratio in LPS-challenged PMVECs. Excessive inflammation is a vital inducer of apoptosis. ADSCs not only modulate the expression of some anti-inflammatory cytokines in some immune cells but also secrete many kinds of cytokines with anti-apoptosis or anti-inflammatory effects, such as IL-10^22-24^. In addition, some apoptosis activators, such as the proapoptotic factor Bax, are highly produced, but Bcl-2 or induced myeloid leukemia cell differentiation protein (MCL-1), two well-known mitochondrial antiapoptotic proteins, are suppressed under LPS treatment. Thus, an imbalance occurs between apoptosis inducers and inhibitors followed by induction of endothelial cell apoptosis. A previous study has shown that downregulation of Bax and upregulation of MCL-1 are detected in resting or IL-8-activated neutrophils upon incubation with MSCs^25^. We deduced that ADSCs might regulate PMVEC apoptosis by mediating the balance between Bax and Bcl-2 under LPS-challenge conditions. Further studies are necessary to confirm the effect and mechanism of ADSCs on the Bcl-2/Bax system in LPS-induced pulmonary microvascular injury.

The therapeutic effects of stem cells have been partly attributed to bioactive paracrine factors. Growth factors are vital effector molecules for ADSC paracrine function in resisting tissue injury and promoting restoration. During LPS-induced lung injury, excessive inflammatory responses ultimately lead to PMVEC apoptosis and tight junction injury. VEGF is essential for maintaining endothelial cell survival and proliferation. In the present study, ADSCs clearly improved the concentration of VEGF in the medium from LPS-triggered PMVECs. On the one hand, ADSCs may secrete VEGF to act as paracrine factors to promote PMVEC survival and reduce apoptosis under LPS treatment. On the other hand, ADSCs are helpful in promoting endothelial cell autocrine VEGF, which is necessary for endothelial survival^26^. In addition, some growth factors have been found to inhibit apoptosis. The addition of vascular endothelial growth factor (VEGF) to the culture medium blocked LPS-induced apoptosis in endothelial cells^27^. Supplement with exogenous VEGF and EGF antagonized the proapoptotic action of staurosporine in primary cultures of human umbilical vein endothelial cells^28^. In addition to VEGF, ADSCs have been shown to release other kinds of growth proteins, including FGF, IGF, and EGF, which are critical for many kinds of cell survival under both physiological and pathological conditions^14,29^. These data account for the improved survival rate of PMVECs upon ADSC incubation.

In the present study, inhibition of autophagy significantly affected the therapeutic effect of ADSCs on LPS-triggered pulmonary microvascular endothelial barrier damage. Based on the vital role of ADSC paracrine effects in protecting against tissue injury, we explored the role of autophagy in regulating ADSC secretion. In the medium from the ADSC and PMVEC coculture system, the inhibition of autophagy in ADSCs markedly reduced the concentration of three essential growth factors, VEGF, FGF, and EGF, under LPS conditions. Additionally, the protective function of ADSCs in PMVECs, including the decrease in tight junction injury and apoptosis rate, was weakened after autophagy inhibition. These findings suggest that autophagy participates, directly or indirectly, in regulating the ADSC secretion process. At the most basic level, the effective paracrine effects of stem cells will occur only if sufficient quantities of vigorous stem cells are activated and mobilized to injured target organs. The stem cells that are activated and mobilized to injured organs will inevitably suffer from the same stress as the target organs. Autophagy has been the vital process in maintaining stem cell survival and vigor under some stress conditions, such as ischemia and hypoxia^30,31^. In the present study, we detected an improved autophagy level in ADSCs cocultured with PMVECs under LPS-challenge conditions. In addition, autophagy has been shown to have a direct regulatory role during stem cell secretion. In cutaneous wound healing, autophagy has been shown to play a role in promoting MSC secretion of VEGF to mediate vascularization^32^. Inhibition of autophagy decreased the TGF-β1 secretion of MSCs, whereas promotion pf autophagy by rapamycin pretreatment caused MSCs to secrete more TGF-β1^33^. It has been reported that autophagy-related genes 5 and 7 may direct lysosomes to fuse with the plasma membrane to participate in lysosomal polarized secretion^34^. In addition, autophagy may directly or indirectly affect the trafficking pathways of some plasma membrane proteins to regulate constitutive and unconventional secretion pathways^35^. These data suggest that autophagy likely participates in the secretion processes of some cytokines from ADSCs. However, in the present research, we did not delineate the regulatory mechanism underlying the effect of autophagy on ADSC paracrine factors, which requires further clarification in future studies.

We showed that LPS challenge greatly increased the paracrine factor level of ADSCs, which was beneficial for maintaining pulmonary microvascular barrier integrity. Inhibition of autophagy weakened paracrine ability and the protective effects of ADSCs in maintaining the integrity of the lung microvascular endothelial barrier under LPS conditions. These results provide new insights into the role and mechanism mediating ADSC paracrine effects in LPS-induced ALI and suggest that the regulation of autophagy might be a potential strategy for modulating the treatment efficacy of ADSCs in lung injury.

## Supplementary information

supplementary materials

Fig. S1

Fig. S2

Fig. S3

Fig. S4

Fig. S5

## References

[1] Johnson ER, Matthay MA (2010). Acute lung injury: epidemiology, pathogenesis, and treatment. J. Aerosol Med. Pulm. Drug Deliv..

[2] Rittirsch D (2008). Acute lung injury induced by lipopolysaccharide is independent of complement activation. J. Immunol..

[3] Frese L, Dijkman PE, Hoerstrup SP (2016). Adipose tissue-derived stem cells in regenerative medicine. Transfus. Med. Hemother. 2016.

[4] Qian J (2016). Protective role of adipose-derived stem cells in Staphylococcus aureus-induced lung injury is mediated by regiiiγ secretion. Stem Cells.

[5] Aboul-Fotouh GI (2015). Therapeutic Effect of Adipose Derived Stem Cells versus Atorvastatin on Amiodarone Induced Lung Injury in Male Rat. Int. J. Stem Cells.

[6] Zhang D (2018). Autophagy maintains the integrity of endothelial barrier in LPS-induced lung injury. J. Cell Physiol..

[7] Dalvi P (2016). Enhanced autophagy in pulmonary endothelial cells on exposure to HIV-Tat and morphine: role in HIV-related pulmonary arterial hypertension. Autophagy.

[8] Fujii S (2012). Insufficient autophagy promotes bronchial epithelial cell senescence in chronic obstructive pulmonary disease. Oncoimmunology.

[9] Ho TT (2017). Autophagy maintains the metabolism and function of young and old stem cells. Nature.

[10] Liu K (2016). ATG3-dependent autophagy mediates mitochondrial homeostasis in pluripotency acquirement and maintenance. Autophagy.

[11] Committee for the Update of the Guide for the Care and Use of Laboratory Animals. (2011). Institute for Laboratory Animal Research. Division on Earth and Life Studies, National Research Council. Guide for the Care and Use of Laboratory Animals.

[12] Smith KM (1997). Prolonged partial liquid ventilation using conventional and high-frequency ventilator techniques: gas exchange and lung pathology in an animal model of respiratory distress syndrome. Crit. Care Med..

[13] Su G (2007). Integrin alphavbeta5 regulates lung vascular permeability and pulmonary endothelial barrier function. Am. J. Respir. Cell Mol. Biol..

[14] Moon KM (2012). The effect of secretory factors of adipose-derived stem cells on human keratinocytes. Int. J. Mol. Sci..

[15] Wu YY (2018). Experimental study on effects of adipose-derived stem cell-seeded silk fibroin chitosan film on wound healing of a diabetic rat model. Ann. Plast. Surg..

[16] Qin LH, Huang W, Mo XA, Chen YL, Wu XH (2015). LPS induces occludin dysregulation in cerebral microvascular endothelial cells via MAPK signaling and augmenting MMP-2 levels. Oxid. Med. Cell Longev..

[17] Lin X, Barravecchia M, Kothari P, Young JL, Dean DA (2016). β1-Na(+),K(+)-ATPase gene therapy upregulates tight junctions to rescue lipopolysaccharide-induced acute lung injury. Gene Ther..

[18] Murakami M (2008). The FGF system has a key role in regulating vascular integrity. J. Clin. Invest..

[19] Bake S, Okoreeh AK, Alaniz RC, Sohrabji F (2016). Insulin-like growth factor (IGF)-I modulates endothelial blood-brain barrier function in ischemic middle-aged female rats. Endocrinology.

[20] Gopalakrishnan S, Hallett MA, Atkinson SJ, Marrs JA (2007). aPKC-PAR complex dysfunction and tight junction disassembly in renal epithelial cells during ATP depletion. Am. J. Physiol. Cell Physiol..

[21] Rashed SM, Patel TB (1991). Regulation of hepatic energy metabolism by epidermal growth factor. Eur. J. Biochem..

[22] Park MJ (2015). Adipose tissue-derived mesenchymal stem cells induce expansion of interleukin-10-producing regulatory B cells and ameliorate autoimmunity in a murine model of systemic lupus erythematosus. Cell Transplant..

[23] Xu L (2016). Hypoxia-induced secretion of IL-10 from adipose-derived mesenchymal stem cell promotes growth and cancer stem cell properties of Burkitt lymphoma. Tumour Biol..

[24] Kuroda K (2015). The paracrine effect of adipose-derived stem cells inhibits osteoarthritis progression. Bmc. Musculoskelet. Disord..

[25] Raffaghello L (2008). Human mesenchymal stem cells inhibit neutrophil apoptosis: a model for neutrophil preservation in the bone marrow niche. Stem Cells.

[26] Domigan CK (2015). Autocrine VEGF maintains endothelial survival through regulation of metabolism and autophagy. J. Cell Sci..

[27] Munshi N, Fernandis AZ, Cherla RP, Park IW, Ganju RK (2002). Lipopolysaccharide-induced apoptosis of endothelial cells and its inhibition by vascular endothelial growth factor. J. Immunol..

[28] Vinci MC (2004). Effect of vascular endothelial growth factor and epidermal growth factor on iatrogenic apoptosis in human endothelial cells. Biochem. Pharmacol..

[29] Deveza L, Choi J, Imanbayev G, Yang F (2013). Paracrine release from nonviral engineered adipose-derived stem cells promotes endothelial cell survival and migration in vitro. Stem Cells Dev..

[30] Asano JS (2017). Intrinsic autophagy is required for the maintenance of intestinal stem cells and for irradiation-induced intestinal regeneration. Cell Rep..

[31] Li C (2017). Rapamycin promotes the survival and adipogenesis of ischemia-challenged adipose derived stem cells by improving autophagy. Cell Physiol. Biochem..

[32] An Y (2018). Autophagy promotes MSC-mediated vascularization in cutaneous wound healing via regulation of VEGF secretion. Cell Death. Dis..

[33] Gao L (2016). Autophagy Improves the Immunosuppression of CD4+ T cells by mesenchymal stem cells through transforming growth factor-β1. Stem Cells Transl. Med..

[34] DeSelm CJ (2011). Autophagy proteins regulate the secretory component of osteoclastic bone resorption. Dev. Cell..

[35] Ponpuak M (2015). Secretory autophagy. Curr. Opin. Cell Biol..

