# Supplementary material for: Autophagy regulates the therapeutic potential of adipose-derived stem cells in LPS-induced pulmonary microvascular barrier damage
Source: Cell Death Dis. 2019 Oct 23;10(11):804. doi: 10.1038/s41419-019-2037-8 (PMC6811543; doi:10.1038/s41419-019-2037-8)
Supplement: Supplementary file 1 — supplementary materials [file 41419_2019_2037_MOESM1_ESM.docx]

**Autophagy regulates the therapeutic potential of adipose-derived stem cells in LPS-induced pulmonary microvascular barrier damage**

**SUPPLEMENTARY MATERIALS AND METHODS**

**Adipose-derived stem cell culture and identification**

We used mouse adipose-derived stem cells (ADSCs, Cloud-Clone Corp., CSI031 Mu01) in in vitro and in vivo experiments. ADSCs were cultured in ADSC complete growth medium. When the cells had grown to 70%–80% confluence, they were harvested with trypsin. After centrifugation, the cells were plated on new culture dishes at approximately 6000 cells/cm^2^. In the present experiment, to determine whether autophagy could influence ADSC characteristics, we performed fluorescence-activated cell sorting analysis by flow cytometry.

**Design and vector preparation of inducible** **lentiviral shRNAs**

To generate shRNA-expressing plasmids, double-stranded oligonucleotides encoding the desired shRNAs were cloned into the AgeI and EcoRI restriction sites of the pLKO-Tet-On vector (Addgene, Inc., Cambridge, MA, USA). Constructs containing an autophagy-related gene (ATG)7-specific shRNA and a scrambled shRNA were designated pLKO-Tet-shRNA-ATG7 and pLKO-Tet-shRNA-Con, respectively.

**Virus production and transfection of target cell lines**

Lentiviruses were generated by cotransfecting 293T cells with 1.5 μg shRNA-encoding plasmids, 1 μg pPAX2 and 0.5 μg pDMG2 (Addgene, Inc. Cambridge, MA) as helper plasmids using Lipofectamine 3000 reagent (Invitrogen Life Technologies) according to the manufacturer's instructions. The growth medium was changed after 8-16 h, and the lentivirus-containing supernatant was harvested after 24, 48 and 72 h.

For transduction, the ADSCs were passaged to 40% confluence the following day. Lentivirus-containing medium was added to the cells with 8 μg/ml polybrene. After 24 h, the viral particle-containing medium was removed and replaced with fresh medium containing 1 μg/ml puromycin. From days 4 to 10, the medium was replaced when necessary, and the cells were evaluated for cytotoxicity under a microscope. Finally, the cells were collected for further experiments and referred to as ADSCs^shRNA-ATG7^ and ADSCs^shRNA-Con^ respectively_._

**Transmission electron microscopy analysis of autophagy ultrastructure**

ADSCs were prefixed with 2.5% glutaraldehyde in 0.1 M PBS at 4°C overnight, postfixed in 1% buffered osmium tetroxide, dehydrated in graded alcohols, embedded in Epon 812, sectioned with an ultramicrotome and stained with uranyl acetate and lead citrate. Autophagosomes in the cells were observed by transmission electron microscopy (TEM) (Philips, CM120). A total of 30 electron microscopic sections were prepared, and the autophagy structures of each group were examined in 200 cells.

**Detection of apoptotic cells in lung tissues**

Cell apoptosis in lung tissues was detected by the TUNEL technique. Four-micrometer thick paraffin sections were deparaffinized and then pretreated with proteinase K for 15 min at room temperature. The TUNEL assay was implemented as described in the body of the manuscript.

**RESULTS**

**Inhibition of ATG7 effectively inhibited the autophagy level of ADSCs**

To assess the role of autophagy in the effects of ADSCs on LPS-induced pulmonary microvascular endothelial barrier damage, we inhibited autophagy by applying shRNAs targeting ATG7. As shown, the expression of ATG7 in ADSCs^shRNA-ATG7^ was much lower than that in ADSCs^shRNA-Con^. Moreover, the shRNA-ATG7 intervention reduced the ratio of microtubule-associated protein light chain (LC3)-II to LC3-I but drove the expression of p62 protein (p62). These data suggest that autophagy was effectively inhibited by lowering ATG7 expression (Fig. S1a).

We observed the characteristics of autophagy-related ultrastructure using transmission electron microscopy. The classic autophagosomes were oval double-membrane structures in which undegraded cargo, such as electron lucent cytoplasm and dense organelles, was detected (Fig. S1b). Statistically, ATG7 inhibition lowered the number of autophagosomes in ADSCs (Fig. S1c).

**Biological characteristics of ADSCs with or without autophagy inhibition**

To determine whether autophagy inhibition affected the biological characteristics of ADSCs, we detected a number of representative surface markers. Flow cytometry analysis demonstrated that ADSCs with or without autophagy inhibition had similar surface markers; the cells were positive for CD90, CD105, CD44 and negative for CD45 (Fig. S2).

**Cocultured ADSCs have no significant effect on the permeability of** **normal living PMVECs**

ADSCs^shRNA-Con^ or ADSCs^shRNA-ATG7^ coculture had not significantly affect the permeability of PMVECs under normal living condition (Fig. S3).

**Inhibition of autophagy weakened the effect of transplanted ADSCs on LPS-induced cells apoptosis in mice lung**

We evaluated cell apoptosis in pulmonary tissues. LPS induced significant cell apoptosis, which was effectively attenuated by transplanted ADSCs. Inhibition of ATG7 expression, however, markedly weakened the efficacy of ADSCs in LPS-induced cell apoptosis (Fig. S4).

**Coculture of ADSCs and normal living PMVECs has no significant influence on the level of VEGF, FGF and EGF in the coculture medium**

Under normal culture conditions, cocultured ADSCs ^shRNA-Con^ or ADSCs ^shRNA-ATG7^ had no significant effect on the levels of VEGF, FGF and EGF in the coculture medium (Supplementary Fig. S5).

Figure Legends

**Fig. S1 ATG7 inhibition lowered the autophagy of ADSCs.** **a** Representative western blot images of autophagy-related proteins LC3 and p62 in the control and ATG7 inhibition groups. **b** Photographs from transmission electron microscopy showing characteristic autophagic ultrastructure in the cells. **c** Quantitative analysis of the number of autophagosomes in different groups. Autophagosomes are indicated by thick white arrows. The results are presented as the mean ± SD (n=3).

**Fig. S2** The effect of autophagy inhibition on the biological characteristics of ADSCs. All ADSCs with or without autophagy inhibition had similar surface markers; the cells were positive for CD90, CD105, CD44 and negative for CD45.

**Fig. S3 Cocultured ADSCs have no significant effect on the permeability of normal PMVECs**

ADSCs^shRNA-Con^ or ADSCs^shRNA-ATG7^ coculture has not significantly affected the permeability of PMVECs under normal living condition.

**Fig. S4 Inhibition of autophagy weakened the effect of transplanted ADSCs on LPS-induced cells apoptosis in mice lung**

LPS induced significant cell apoptosis, which was effectively attenuated by transplantation of ADSCs. Inhibition of ATG7 expression, however, markedly weakened the efficacy of ADSCs in LPS-induced cells apoptosis.

**Fig. S5 Coculture of ADSCs and normal PMVECs has no significant effect on the level of VEGF, FGF and EGF in the coculture medium**

Under normal culture condition, cocultured ADSCs ^shRNA-Con^ or ADSCs ^shRNA-ATG7^ has no significant effect on the levels of VEGF, FGF and EGF in the coculture medium.
