# Supplementary figures and images for: Autophagy regulates the therapeutic potential of adipose-derived stem cells in LPS-induced pulmonary microvascular barrier damage
Source: Cell Death Dis. 2019 Oct 23;10(11):804. doi: 10.1038/s41419-019-2037-8 (PMC6811543; doi:10.1038/s41419-019-2037-8)

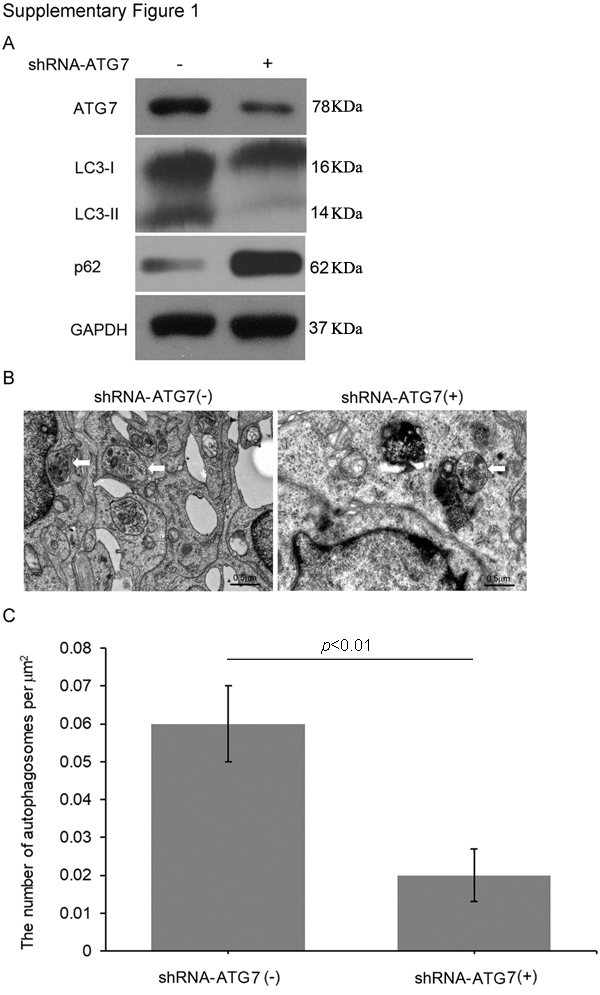

Supplement: Supplementary file 2 — Fig. S1 [file 41419_2019_2037_MOESM2_ESM.tif]

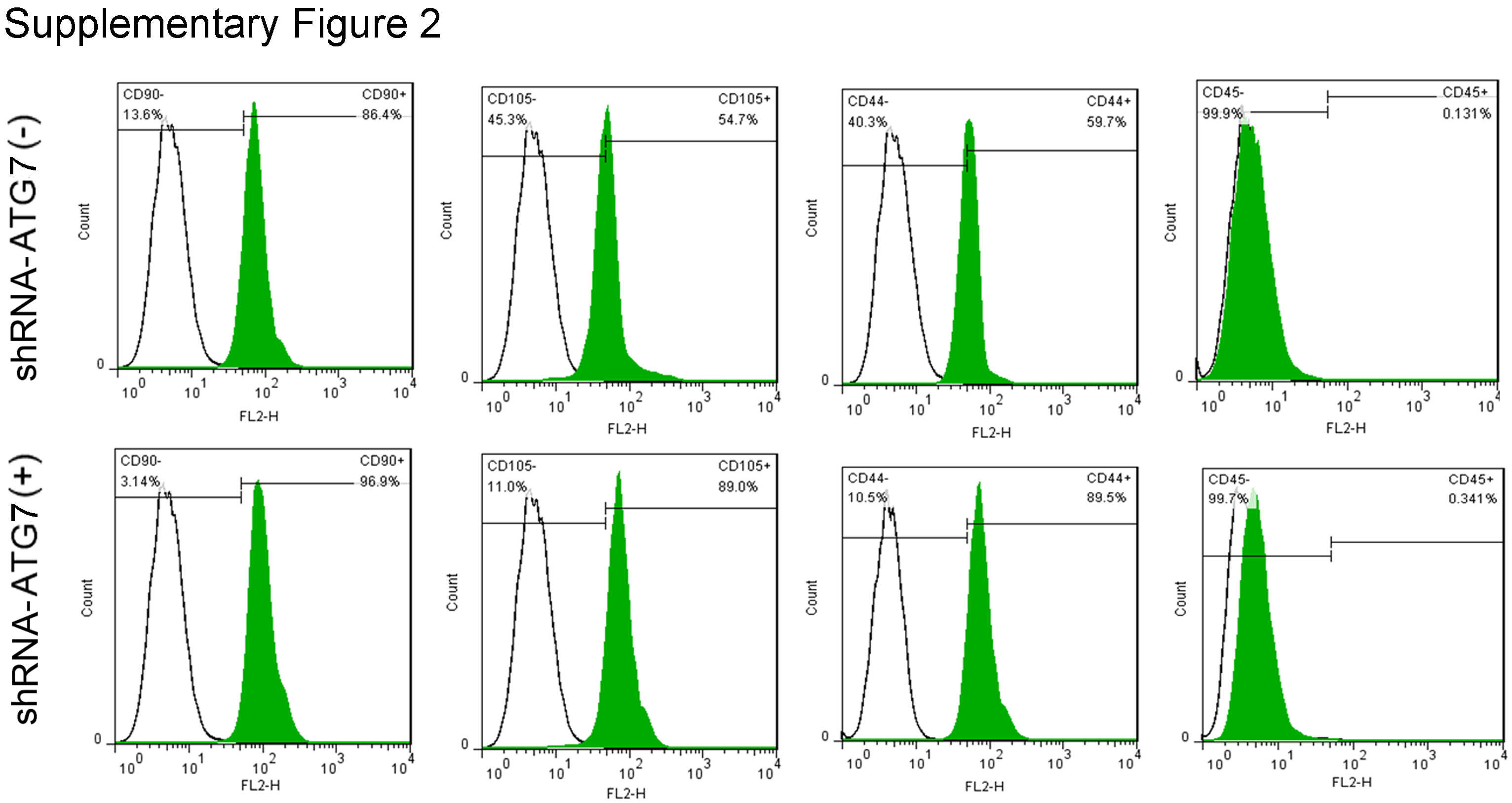

Supplement: Supplementary file 3 — Fig. S2 [file 41419_2019_2037_MOESM3_ESM.tif]

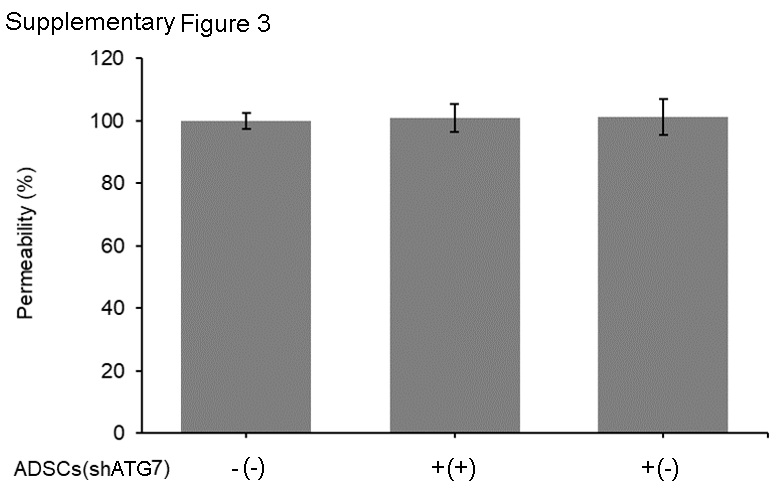

Supplement: Supplementary file 4 — Fig. S3 [file 41419_2019_2037_MOESM4_ESM.tif]

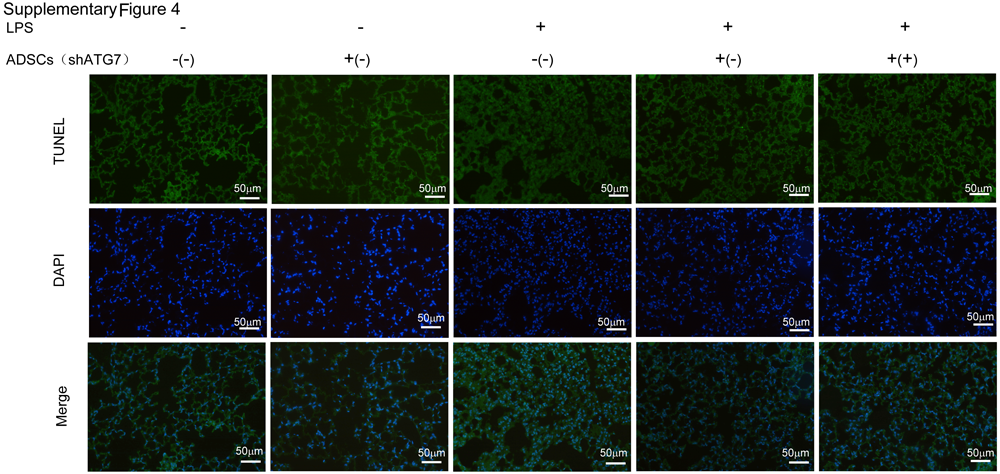

Supplement: Supplementary file 5 — Fig. S4 [file 41419_2019_2037_MOESM5_ESM.tif]

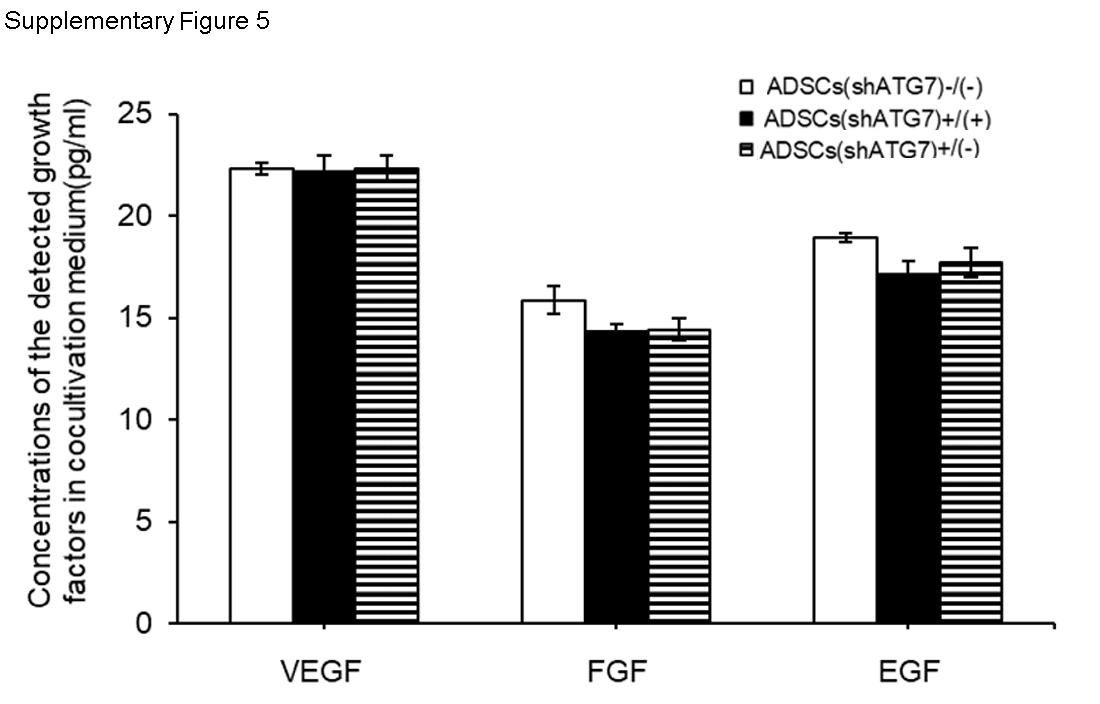

Supplement: Supplementary file 6 — Fig. S5 [file 41419_2019_2037_MOESM6_ESM.tif]
